# Utilization and efficacy of second-line targeted therapy in metastatic renal cell carcinoma: data from a national registry

**DOI:** 10.1186/s12885-017-3901-5

**Published:** 2017-12-21

**Authors:** Radek Lakomy, Alexandr Poprach, Zbynek Bortlicek, Bohuslav Melichar, Renata Chloupkova, Rostislav Vyzula, Milada Zemanova, Katerina Kopeckova, Marek Svoboda, Ondrej Slaby, Igor Kiss, Hana Studentova, Jaroslav Juracek, Ondrej Fiala, Jindrich Kopecky, Jindrich Finek, Ladislav Dusek, Karel Hejduk, Tomas Buchler

**Affiliations:** 1 Department of Comprehensive Cancer Care and Faculty of Medicine, Masaryk Memorial Cancer Institute and Masaryk University, Brno, Czech Republic; 2 Institute of Biostatistics and Analyses, Faculty of Medicine, Masaryk University, Brno, Czech Republic; 3 Department of Oncology, Palacky University Medical School and Teaching Hospital, I.P. Pavlova 6, 775 20 Olomouc, Czech Republic; 4 Department of Oncology, First Faculty of Medicine, Charles University and General University Hospital, U Nemocnice 499/2, 128 08 Prague, Czech Republic; 5 Department of Oncology, Second Faculty of Medicine, Charles University and Motol University Hospital, Prague, Czech Republic; 6 Department of Oncology, Charles University and University Hospital, Svobody 80, 304 60 Pilsen, Czech Republic; 7 Department of Oncology, Hradec Králové University Hospital and Faculty of Medicine, Charles University, Hradec Králové, Sokolská 581, 50005 Hradec Králové, Czech Republic; 8 Department of Oncology, First Faculty of Medicine, Charles University and Thomayer Hospital, Videnska 800, 140 59 Prague, Czech Republic

**Keywords:** Renal cell carcinoma, Therapy, Sunitinib, Sorafenib, Everolimus, Pazopanib

## Abstract

**Background:**

It is well known that patient characteristics and survival outcomes in randomized trials may not necessarily be similar to those in real-life clinical practice. The aim of the present study was to analyse second line treatment strategies in the real-world practice and to estimate the outcomes of patients treated with second-line targeted therapy for metastatic renal cell carcinoma (mRCC).

**Methods:**

This is a retrospective, registry-based study using data from the national registry of targeted therapies for mRCC. The RENIS registry contains data on 3049 patients who started the therapy with at least one targeted agent before 31 December, 2014. Of these patients, 1029 had a record of at least two different targeted therapies and sufficient data for analysis. Survival analysis was carried out using the Kaplan-Meier method. Statistical significance of differences in survival between subgroups was assessed using the log-rank test.

**Results:**

The median overall survival from the start of second-line treatment was 17.0 months (95% confidence interval [CI] 14.5–19.5 months), 17.1 months (95% CI 14.5–19.8), and 15.4 months (95% CI 11.0–19.7) for second-line everolimus, sorafenib, and sunitinib, respectively. Patients receiving second-line everolimus were older at the start of second-line treatment, more likely to have metachronous disease, and less likely to be previously treated with cytokines or to continue to third-line treatment than patients treated with second-line sunitinib or sorafenib. Progression-free survival (PFS) correlated with PFS on first-line treatment only for everolimus.

**Conclusions:**

In this retrospective study, no significant differences in survival were observed between the cohorts treated with different second-line agents including everolimus, sorafenib, and sunitinib.

## Background

Current options for first-line treatment of metastatic renal cell cancer (mRCC) include agents targeting the vascular endothelial growth factor (VEGF) signalling pathway sunitinib, pazopanib, and bevacizumab (administered in combination with interferon) as well as inhibitor of mammalian target of rapamycin (mTOR) temsirolimus. The standard second-line treatment options are even wider and include mTOR inhibitor (mTORi) everolimus, VEGF inhibitor (VEGFi) axitinib and three new drugs introduced to the clinical practice in 2016, nivolumab, cabozantinib, and lenvatinib. VEGF pathway inhibitors such as sunitinib, sorafenib, or pazopanib may be considered as options for second-line treatment in specific cases [1].

The use of everolimus and axitinib is based on prospective randomized phase III trials: Everolimus was shown to prolong progression-free survival (PFS) compared to placebo in second and higher lines of systemic therapy of mRCC patients, but the overall survival (OS) was not significantly extended, probably due to cross-over from placebo to everolimus arm [2]. Axitinib was also demonstrated to prolong PFS compared to sorafenib, but, again, OS prolongation compared to sorafenib was not statistically significant [3].

It is well known that patient characteristics and survival outcomes in randomized trials may not necessarily be similar to those in real-life patients [4]. Moreover, three more therapeutic options have emerged very recently in the second line therapy of mRCC, including cabozantinib, nivolumab and lenvatinib. In face of such many available treatment options, it is important to re-evaluate the results obtained with currently available drugs in this setting.

The objective of the present retrospective, registry-based study was to analyse second line treatment strategies in the real-world practice and to estimate the outcomes of patients treated with second-line targeted therapy for mRCC.

## Methods

### Patients

The RENIS registry contains data on patients who started the therapy with at least one targeted agent for mRCC [5, 6]. All patients who started a second-line targeted therapy before 31 December, 2014 were included in this study. Previous cytokine therapy was not considered as a treatment line for the purpose of the present analysis The date was selected in order to ensure a meaningful follow-up period. Only patients with recorded progression after the first-line therapy were included. Prior analyses on some aspects of second-line therapies have been previously reported based on RENIS registry [7–9]. The RENIS registry and retrospective analyses of registry data have been approved by the Ethics Committee, Masaryk Memorial Cancer Institute, Brno, Czech Republic.

### Data source

The RENIS registry contains data on approximately 95% of all Czech patients with mRCC treated with targeted therapy of any line. In the Czech Republic the reimbursement of targeted therapies is restricted to comprehensive cancer care centres. The data in the RENIS registry are stored in an anonymised form and updated twice a year. Baseline patient characteristics as well as the information on previous treatment such as surgery, radiotherapy, or immunotherapy are recorded. Vital signs, biochemical and haematological parameters are recorded prior to the initiation of targeted therapy of any line, and the best treatment response, adverse events, PFS and OS are evaluated throughout [5]. Treatment response is assessed using the Response Evaluation Criteria In Solid Tumors (RECIST) 1.1 criteria and treatment toxicity with the National Cancer Institute Common Terminology Criteria for Adverse Events (NCI-CTCAE) Version 3.0 [10, 11].

### Statistical analysis

Frequency analysis and summary statistics were used to characterise the sample data set. Statistical significance of differences between age subgroups in categorical parameters was assessed using the Fisher’s exact test. The Kruskal-Wallis test was used for continuous variables.

PFS was defined as the time from the initiation of second-line therapy to the date of first documented progression or death due to any cause and OS as the time from the onset of second-line therapy to death due to any cause. PFS and OS were analysed using the Kaplan-Meier method. Statistical significance of differences in time to survival events between subgroups was assessed using the log-rank test. All statistical tests were performed at a significance level of α = 0.05.

## Results

### Sequential treatment strategies

The RENIS registry contains data on 3049 patients who started the therapy with at least one targeted agent before 31 December, 2014. The number of patients in the RENIS registry who completed first-line treatment was 2473. Of these patients, 1253 (50.7%) had a record of at least two different targeted therapies. In total, 1029 patients were eligible for this analysis based on the above criteria. Baseline patient characteristics are summarised in Table 1. Interestingly, patients receiving second-line everolimus were older at the start of second-line treatment, more likely to have metachronous disease, and less likely to be previously treated with cytokines or to continue to third-line treatment than patients treated with second-line sunitinib or sorafenib. Sunitinib-everolimus was the most frequently used sequence (*n* = 390, 38%), followed by sunitinib-sorafenib (*n* = 232, 23%), sorafenib-sunitinib (*n* = 139, 14%) and sorafenib-everolimus (*n* = 93, 9%) (Table 2).

**Table 1:** Baseline characteristics of the patients

	Everolimus (*n* = 520)	Sorafenib (*n* = 240)	Sunitinib (*n* = 228)	*p*-value*
Gender, *n* (%)				
Male	392 (75.4)	179 (74.6)	169 (74.1)	0.914
Female	128 (24.6)	61 (25.4)	59 (25.9)	
Age at diagnosis [years]				
Median (range)	60 (33–81)	57 (33–78)	60 (33–80)	0.009
Metastatic disease, *n* (%)				
Metachronous	264 (55.8)	105 (49.5)	85 (42.7)	0.007
Synchronous	209 (44.2)	107 (50.5)	114 (57.3)	
Unknown	47	28	29	
Histology, n (%)				
Clear-cell carcinoma	490 (94.2)	227 (94.6)	213 (93.4)	0.938
Papillary carcinoma	25 (4.8)	10 (4.2)	13 (5.7)	
Other	5 (1.0)	3 (1.3)	2 (0.9)	
MSKCC^a^	520 (100.0)	240 (100.0)	228 (100.0)	0.058
Good prognosis	185 (35.6)	89 (37.1)	87 (38.2)	
Intermediate prognosis	316 (60.8)	135 (56.3)	122 (53.5)	
Poor prognosis	19 (3.7)	16 (6.7)	19 (8.3)	
Previous nephrectomy, *n* (%)	444 (85.4)	216 (90.0)	189 (82.9)	0.072
Previous cytokines, *n* (%)	180 (34.6)	158 (65.8)	134 (58.8)	< 0.001
Age at onset of second-line therapy [years]				
Median (range)	65 (37–83)	62 (35–83)	62 (34–82)	< 0.001
ECOG PS at onset of second-line therapy, *n* (%)				
0	109 (27.8)	37 (21.5)	36 (23.7)	0.084
1	265 (67.6)	117 (68.0)	105 (69.1)	
2	17 (4.3)	18 (10.5)	11 (7.2)	
3	1 (0.3)	0 (0.0)	0 (0.0)	
Unknown	128	68	76	
Reason for treatment discontinuation, *n* (%)				
Progression or death	349 (78.3)	177 (76.3)	151 (72.9)	–
Adverse event	33 (7.4)	28 (12.1)	32 (15.5)	
Other	64 (14.3)	27 (11.6)	24 (11.6)	
Treatment duration [months]				
Mean (25–75 percentile)	6.1 (2.7–8.6)	7.1 (2.1–8.0)	7.1 (2.6–9.7)	–
Third-line targeted therapy, *n* (%)	79 (15.2)	80 (33.3)	61 (26.8)	–
Fourth-line targeted therapy, *n* (%)	2 (0.4)	3 (1.3)	4 (1.8)	–

*MSKCC* Memorial Sloan Kettering Cancer Center, *ECOG PS* performance status according to *Eastern Cooperative Oncology Group*

^*^Fisher exact test or Kruskal-Wallis test

^a^MSKCC score calculated at the start of first-line therapy

**Table 2:** First and second-line targeted drugs used in the present cohort

First targeted therapy	Second targeted therapy						
	Everolimus (*n* = 520)	Sorafenib (*n* = 240)	Sunitinib (n = 228)	Axitinib (*n* = 29)	Pazopanib (*n* = 10)	Temsirolimus (*n* = 1)	Bevacizumab + IFN (*n* = 1)
Bevacizumab + IFN	5 (1.0%)	4 (1.7%)	19 (8.3%)	0 (0.0%)	7 (70.0%)	0 (0.0%)	0 (0.0%)
Sorafenib	93 (17.9%)	0 (0.0%)	139 (61.0%)	0 (0.0%)	0 (0.0%)	0 (0.0%)	0 (0.0%)
Sunitinib	390 (75.0%)	232 (96.7%)	0 (0.0%)	28 (96.6%)	3 (30.0%)	1 (100.0%)	1 (100.0%)
Temsirolimus	0 (0.0%)	2 (0.8%)	21 (9.2%)	0 (0.0%)	0 (0.0%)	0 (0.0%)	0 (0.0%)
Pazopanib	32 (6.2%)	2 (0.8%)	49 (21.5%)	1 (3.4%)	0 (0.0%)	0 (0.0%)	0 (0.0%)

*IFN* interferon-α

### Treatment outcomes and toxicity

Five hundred sixty-six patients had died before the date of data cut-off on 14 March, 2016. The median follow-up of surviving patients in the present cohort was 29.2 months from the start of first-line targeted therapy. Treatment outcomes were analysed for the three most commonly used second line agents, i.e. everolimus, sunitinib, and sorafenib.

Before the data cut-off date treatment was discontinued in 446 (85.8%), 232 (96.7%), and 207 (90.8%) patients treated with everolimus, sorafenib and sunitinib, respectively. Disease progression or death was the most frequent reason for treatment discontinuation (78.3%, 76.3% and 72.9% of patients treated with everolimus, sorafenib, and sunitinib, respectively). By the time of data cut-off, third-line treatment was started in 79 patients and a fourth-line therapy in two patients treated with everolimus in the second line, 80 and three patients, respectively, treated with second line sorafenib, and 61 and four patients, respectively, treated with second line sunitinib.

**Fig. 1:**
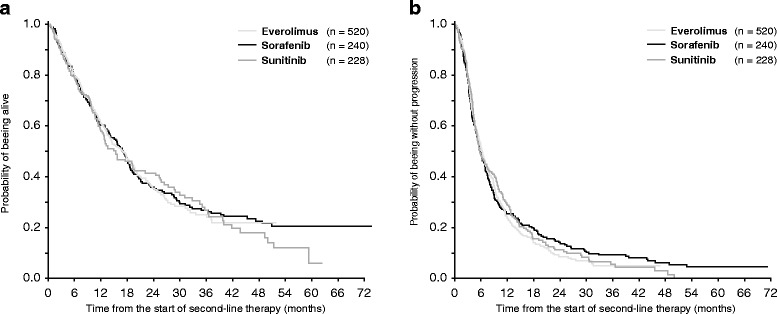
Overall survival (**a**) and progression-free survival (**b**) from the start of second-line targeted therapy

The response rates, PFS and OS are shown in Table 3 and Fig. 1. No significant differences in survival were observed between the cohorts treated with different second-line agent. Table 3 also shows outcomes of the four most common sequences of targeted agents. Similar results were also obtained for the patients treated with cytokines prior to first-line targeted therapy (data not shown).

**Table 3:** Overall survival and progression-free survival from the start of second-line therapy for individual drugs and from the start of first-line therapy for the most common treatment sequences

Second-line targeted drug	*n*	Median OS (95% CI)	Median PFS (95% CI)	Overall response rate, *n* (%) ^a^	Disease control rate *n* (%)^a^
Everolimus	520	17.0 months (14.5–19.5)	6.3 months (5.6–6.9)	30 (6.7)	201 (45.1)
Sorafenib	240	17.1 months (14.5–19.8)	5.8 months (4.7–6.8)	23 (9.9)	113 (48.7)
Sunitinib	228	15.4 months (11.0–19.7)	5.7 months (4.4–7.0)	25 (12.1)	73 (35.3)
Sunitinib → everolimus	390	37.2 months (31.5–42.9)	22.7 months (19.5–26.0)^b^	32 (8.2)	176 (45.1)
Sunitinib → sorafenib	232	32.7 months (27.1–38.2)	19.0 months (15.8–22.3 ^b^	24 (10.3)	119 (51.3)
Sorafenib → sunitinib	139	31.8 months (25.8–37.7)	18.5 months (16.2–20.7)^b^	19 (13.7)	54 (38.8)
Sorafenib → everolimus	93	32.2 months (26.0–38.3)	21.4 months (18.0–24.9)^b^	3 (3.2)	52 (55.9)

*95%CI* 95% confidence intervals, *OS* overall survival, *PFS* progression-free survival

^a^Data for best response assessment were available for 446, 232, and 207 patients treated with everolimus, sorafenib, and sunitinib, respectively

^b^PFS for sequences was the time from first-line treatment initiation to the date of documented progression on the second-line therapy

Table 4 shows PFS and OS for prognostic subgroups according to the International Metastatic Renal Cell Carcinoma Database Consortium (IMDC) model and according to the Memorial Sloan Kettering Cancer Center (MSKCC) model. Although the registry only contained data for calculation of these predictors at the start of first-line targeted therapy, both models were predictive for outcomes of second-line therapy.

**Table 4:** Overall survival and progression-free survival from the start of second-line targeted therapy according to MSKCC and IMDC risk groups (patients with evaluable MSKCC score, *n* = 1029; patients with evaluable IMDC score, *n* = 540)

Prognostic score	*n*	Median OS (months) (95% CI)	Median PFS (months) (95% CI)
IMDC good	178	20.2 (13.6–26.8)	6.9 (5.0–8.8)
IMDC intermediate	314	17.0 (13.1–20.9)	5.8 (5.1–6.5)
IMDC poor	48	11.5 (5.3–17.7)	3.7 (2.8–4.6)
MSKCC good	378	19.2 (16.3–22.0)	7.3 (6.1–8.6)
MSKCC intermediate	596	15.7 (13.5–17.9)	6.0 (5.4–6.5)
MSKCC poor	55	5.5 (3.1–7.8)	3.7 (3.0–4.3)

*IMDC* International Metastatic Renal Cell Carcinoma Database Consortium, *MSKCC* Memorial Sloan Kettering Cancer Center, *OS* overall survival, *PFS* progression-free survival

For patients with non-clear cell mRCC (*n* = 58) the PFS was 4.1 months (95% confidence interval [CI] 1.1–7.2), 4.1 months (95% CI 1.1–7.2), and 17.8 months (95% CI 0.1–38.2) and the OS 11.4 months (95% CI 4.2–18.6), 14.3 months (95% CI 8.2–20.3), and 36.5 months (95% CI 0.1–73.0) for patients treated with everolimus (*n* = 30), sorafenib (*n* = 13), and sunitinib (*n* = 15), respectively.

Of all patients who discontinued therapy, adverse events were the reason for treatment discontinuation in 7.4%, 12.1%, and 15.5% of patients treated with second-line everolimus, sorafenib, and sunitinib, respectively (Table 1). Incidences of serious adverse events reported during second-line therapy are shown in Table 5.

**Table 5:** Serious adverse events reported during second-line therapy

	Everolimus (*n* = 520)	Sorafenib (*n* = 240)	Sunitinib (*n* = 228)
Serious adverse events, n (%)	24 (4.6)	31 (12.9)	29 (12.7)
Types of serious adverse events^a^, *n* (%)			
Gastrointestinal	3 (0.6)	8 (3.3)	8 (3.5)
Haematologic	2 (0.4)	3 (1.3)	8 (3.5)
Cardiovascular	1 (0.2)	3 (1.3)	3 (1.3)
Metabolic	2 (0.4)	6 (2.5)	1 (0.4)
Dermatologic	2 (0.4)	9 (3.8)	1 (0.4)
Respiratory	8 (1.5)	0 (0.0)	2 (0.9)
Fatigue	2 (0.4)	2 (0.8)	1 (0.4)
Other	5 (1.0)	6 (2.5)	5 (2.2)

^a^One patient can have more serious adverse events

### Association between PFS on first and second targeted therapy

To test the hypothesis that PFS on second-line drug is associated with the PFS on first-line therapy, an arbitrary PFS threshold of 9 months (corresponding to the median PFS of 10 months reported for first-line targeted agents) was set for the first-line targeted therapy. An association between PFS on the first-line and second-line therapy was observed only for second-line everolimus, but not for sunitinib or sorafenib. Similarly, OS was associated with longer PFS on the first-line therapy only in patients treated with second-line everolimus (Table 6).

**Table 6:** Overall survival and progression-free survival from the start of second-line targeted therapy according to progression-free survival on first-line targeted therapy

Second-line targeted therapy	*n*	OS		PFS	
		Median (95% CI)	*P*	Median (95% CI)	*P*
Everolimus					
PFS1 < 9 months	230	12.7 months (10.5–14.9)	< 0.001	5.0 months (3.9–6.1)	< 0.001
PFS1 ≥ 9 months	290	19.7 months (16.4–23.0)		7.3 months (5.9–8.8)	
Sorafenib					
PFS1 < 9 months	123	13.8 months (7.9–19.7)	0.083	5.4 months (4.1–6.7)	0.628
PFS1 ≥ 9 months	117	17.5 months (14.6–20.4)		6.2 months (5.0–7.4)	
Sunitinib					
PFS1 < 9 months	136	12.9 months (5.7–20.1)	0.171	5.0 months (4.0–6.0)	0.571
PFS1 ≥ 9 months	92	15.7 months (9.5–22.0)		6.9 months (4.0–9.7)	

*95% CI* 95% confidence interval, *OS* overall survival, *PFS* progression-free survival, *PFS1* progression-free survival on first-line targeted therapy

## Discussion

The present study documents similar survival achieved with different second-line options for mRCC in the real clinical practice. It also shows declining prescription rates for sorafenib as a second-line option and gradual adoption of the VEGFi-VEGFi strategy as opposed to VEGFi-mTOR strategy in the last years.

The outcomes of patients from the national RENIS registry are comparable with the efficacy data reported from the prospective second-line trials in patients with mRCC. A correlation between the duration of PFS on first-line therapy and second-line treatment was observed in patients treated with second-line everolimus.

The present retrospective study was primarily descriptive, and no conclusions regarding the relative efficacy of the second-line agents can be inferred from these results. Obviously, such data can be obtained only in prospective randomized studies.

Everolimus, sorafenib and sunitinib have previously been studied in prospective trials. In the RECORD-1 study, patients pre-treated with one line of anti-VEGF therapy had PFS of 5.4 months on everolimus [2]. Median PFS for second-line everolimus in the CHANGE study was 6.9 months [12]. These results correspond to those of in RENIS database (6.3 months).

Efficacy of second-line sorafenib and sunitinib in mRCC patients was studied in the prospective, randomised SWITCH trial that compared sunitinib-sorafenib and sorafenib-sunitinib sequences. Median total PFS was 14.9 months in the sunitinib-sorafenib arm and 12.5 months in the sorafenib-sunitinib arm; median PFS of second-line treatment was 2.8 months for sunitinib and 5.4 months for sorafenib [13]. Similar findings from a retrospective study were published earlier by our group based on the RENIS registry [7]. Median PFS for sorafenib in a randomised prospective AXIS study comparing second-line sorafenib with axitinib was 3.4 months compared to 4.8 months for axitinib in patients after previous sunitinib therapy [3]. In the present analysis, median PFS for sorafenib and sunitinib was 5.8 and 5.7 months, respectively, and these findings based on real-life patients are comparable to the results of the above prospective studies.

In addition to prospective trials, several recent studies based on real-world data have shed light not only on prescription patterns of targeted agents, but also on the outcomes of various sequencing strategies that are difficult if not impossible to evaluate in prospective randomised trials.

Pal et al. studied a cohort of 696 patients treated with everolimus as the second targeted agent, comparing outcomes of patients treated with first-line sunitinib or sorafenib and patients who received pazopanib, a drug with no second-line treatment established based on randomised trials. The outcomes of patients treated with sunitinib or sorafenib and pazopanib were similar [14].

The IMDC have done much to further our understanding of treatment sequencing in mRCC. The IMDC model has been validated for predicting the outcomes of second-line treatment [15].

In a cohort from a community-academic registry of mRCC patients Harrison et al. showed that various sequencing patterns may translate into different outcomes, in particular regarding the first-line therapy with mTORi which was also associated with worse survival in a prospective trial [16, 17].

The results of observational studies investigating the VEGFi-VEGFi versus VEGFi-mTORi sequences are inconclusive due to significant heterogeneity in the observed effect of second-line therapies and potential bias [18–20]. The only randomised study of second-line therapy directly comparing VEGFi with mTORi is the recently published METEOR trial. In the METEOR trial, PFS was longer with cabozantinib compared to everolimus in patients who had progressed after VEGFi [21]. However, cabozantinib is also a c-Met inhibitor in contrast to other VEGFi such as sunitinib, pazopanib, sorafenib, and axitinib. The results obtained with cabozantinib may not be generalizable to other anti-VEGF agents as c-Met pathway is important in the development of secondary resistance to VEGFi.

Recently, an analysis of the IMDC database suggested that the change in the IMDC prognostic score can be predictive for response to second-line therapies. Patients who had deteriorated from favourable to intermediate prognosis while on first-line VEGFi benefited from switch to mTORi [22].

It remains to be seen whether the issue of VEGFi-mTORi versus VEGFi-VEGFi sequence will become moot with the arrival of agents with different mechanism of action for second-line therapy, in particular nivolumab [23]. The advent of immunotherapy will undoubtedly add new complexities to the discussion of mRCC second line-treatment strategy. First, PFS may not be a meaningful parameter in patients treated with immune checkpoint inhibitors like nivolumab. Moreover, immunotherapy may result in long-term disease control in a substantial proportion of patients, something that is unusual with other targeted agents.

The question whether the efficacy of first-line treatment can be used to guide the selection of the second-line therapy has been studied by several groups in retrospective studies. Al-Marrawi et al. did not detect any association between PFS on the first- and second-line VEGFi [24]. In an earlier study based on RENIS registry we also addressed this issue with similar conclusion for sunitinib-sorafenib or reverse sequences [7]. However, in subsequent analysis we observed an association between PFS on first-line VEGFi with PFS on second-line everolimus, a finding confirmed in the present report on an updated cohort [9]. Recently, Vogelzang et al. compared second-line therapy with everolimus and axitinib. No significant differences were seen in PFS or OS and PFS on first-line anti-VEGF agent was not useful for second-line treatment selection [25]. This topic was recently addressed by several studies that point out that patients primarily refractory to VEGFi will have poor prognosis regardless of second-line strategies [26, 27]. It remains to be determined whether the association observed between PFS on the first or second line therapies reflects susceptibility of the tumour to targeted agents or rather a more indolent course of disease.

The proportion of patients who received second-line treatment (50.6%) in the present study is consistent with most previously published observations, such as the study of Levy et al. (52%) and Beisland et al. (45% of the 2011 cohort) [28, 29]. In the largest, IMDC registry-based study of 2106 patients with the median follow-up of 36 months, 43% received second line therapy [30]. On the other hand, only 15% of patients from the British RECCORD database received second line-therapy [31].

The present retrospective study has several limitations. Pazopanib was not available in the Czech Republic before 2010 and was subject to reimbursement restrictions afterwards. Everolimus was generally not reimbursed when administered after pazopanib until 2014. As a consequence, pazopanib-everolimus sequence was used in only 32 patients in the RENIS database. PFS and OS assessment for axitinib that currently represents a prominent second-line agent was not possible due to the short duration of its use in the Czech Republic, with only 29 patients were included in the database fulfilling the inclusion criteria for the present study. Due to the retrospective character of the present study, there is obviously a risk of selection bias resulting in imbalances between the subgroups and underreporting of adverse events. Moreover, the timing of disease assessment with imaging differed among centres, and there was no central review of the response.

Some trends observed in the present study are best explained by changing availability of reimbursed drugs. No further targeted therapies were reimbursed after everolimus, explaining the lower uptake of third-line treatments in these patients. The decline in utilisation of everolimus in 2012 is likely related to the arrival of pazopanib. According to reimbursement rules at the time, second-line everolimus was not allowed after pazopanib and the patients had to have another line of VEGFRi, mostly sunitinib.

## Conclusion

We report here national registry-based data on the efficacy and safety of everolimus, sorafenib, and sunitinib as second-line therapy of mRCC that are comparable with the results of prospective randomized clinical trials. Currently, no single agent can be regarded as unambiguously superior in this setting. International guidelines should be observed and treatment should be selected in conformity to individual patient clinical characteristics.

## Abbreviations

CI: Confidence intervals
ECOG PS: Performance status according to *Eastern Cooperative Oncology Group*
IFN: Interferon-α
IMDC: International Metastatic Renal Cell Carcinoma Database Consortium
MRCC: Metastatic renal cell cancer
MSKCC: Memorial Sloan Kettering Cancer Center
MTOR: Mammalian target of rapamycin
Mtori: mammalian target of rapamycin inhibitor
NCI-CTCAE: National Cancer Institute Common Terminology Criteria for Adverse Events
OS: The overall survival
PFS: Progression-free survival
PFS1: Progression-free survival on first-line targeted therapy
RECIST: Response Evaluation Criteria In Solid Tumors
RECORD-1: REnal Cell cancer treatment with Oral RAD001 given Daily
VEGF: Vascular endothelial growth factor
VEGFi: Vascular endothelial growth factor inhibitor
